# Positive Psychological Factors Are Associated With Better Spiritual Well-Being and Lower Distress in Individuals With Skin Diseases

**DOI:** 10.3389/fpsyg.2020.552764

**Published:** 2020-10-06

**Authors:** Luca Iani, Rossella Mattea Quinto, Piero Porcelli, Andrea-René Angeramo, Andrea Schiralli, Damiano Abeni

**Affiliations:** ^1^Department of Human Sciences, European University of Rome, Rome, Italy; ^2^Department of Psychological, Health and Territorial Sciences, University of “G. d’Annunzio” Chieti–Pescara, Chieti, Italy; ^3^IDI-IRCCS, Rome, Italy

**Keywords:** skin diseases, sense of coherence, emotion regulation, positivity, spiritual well-being, psychological distress, expressive suppression

## Abstract

In this study we examined whether aspects of positive functioning [reappraisal, sense of coherence (SOC), and positivity] were associated with spiritual well-being and psychological distress after controlling for negative functioning (skin-related symptoms, type of disease, and expressive suppression) in individuals with skin diseases. We also examined whether negative functioning aspects were linked to spiritual well-being and distress when controlling for aspects of positive functioning. The study used a cross-sectional design with a sample of 192 individuals with psoriasis and systemic sclerosis (SSc) (*M*_*age*_ = 51.6 years, *SD* = 16.5). Two hierarchical multiple regressions were conducted to determine whether spiritual well-being and psychological distress were accounted for by skin-related symptoms, type of disease, emotion regulation strategies, SOC, and positivity. Positivity was the most important contributor to better spiritual well-being, followed by both the comprehensibility/manageability and meaningfulness SOC subscales, after controlling for the other variables. High skin-related symptoms and expressive suppression were associated with lower psychological distress, whereas high SOC and reappraisal correlated with higher psychological distress. The findings of this study pave the way for further research on how SOC and positivity may reduce the effects of both skin-related symptoms and emotion dysregulation and facilitate spiritual well-being of individuals with skin diseases. Interventions aimed to enhance inner resources of these individuals and help them to find a meaning in their experience of skin disease might reduce psychological distress and improve spiritual well-being. Our findings suggest that healthcare professionals should consider positive functioning aspects in future interventions for individuals with skin diseases.

## Introduction

Psoriasis is a chronic, inflammatory, immune-mediated, skin disease that greatly affects health-related quality of life (HRQOL; Augustin and Radtke, 2014). Psoriasis is associated with negative psychosocial outcomes, including lower psychological well-being and higher psychological distress (Offidani et al., 2016), psychiatric morbidity (Pompili et al., 2017), cognitive impairment (Innamorati et al., 2018), and impulse control difficulties (Almeida et al., 2017). Systemic sclerosis (SSc) is an autoimmune connective tissue disease characterized by progressive vasculopathy and fibrosis of skin and internal organs (Varga et al., 2017). A consistent body of evidence showed that SSc is associated with quality of life impairment (Park et al., 2019), fatigue (Sandusky et al., 2008), and depression and anxiety (Baubet et al., 2011). Moreover, individuals with SSc reported both higher psychological distress (Hyphantis et al., 2007a) and lower meaning and sense (Pilch et al., 2016) than healthy individuals.

Physical symptoms severity is an important clinical aspect of disability that may cause psychological distress in dermatological patients. Some of the most important somatic conditions that affect quality of life in patients with skin diseases are itch and fatigue, which were reported by more than 50% of all patients (Verhoeven et al., 2007). Different physical symptoms including itching, irritation, and burning, were prevalent in patients with psoriasis and with concurrent psychological distress (Sampogna et al., 2004a). Moreover, higher levels of fatigue best predicted psychological distress in patients with psoriasis and atopic dermatitis (Evers et al., 2005).

Emotion regulation is also related to quality of life in skin diseases. Previous studies suggested a strong association between skin diseases and emotion dysregulation (Innamorati et al., 2016; Quinto et al., 2019). Emotion regulation is the process that helps reducing, maintaining, or increasing one or more aspects of emotion (Werner and Gross, 2010). Two of the most commonly used emotion regulation strategies are expressive suppression and cognitive reappraisal. Expressive suppression refers to the “efforts to inhibit ongoing emotion-expressive behavior” (Werner and Gross, 2010, p. 29), whereas cognitive reappraisal refers to changing the way in which individuals appraise a situation to modify its emotional significance. Emotional suppression was found to be a dysfunctional emotion regulation strategy, while cognitive reappraisal was found to be adaptive (Werner and Gross, 2010). Although research has demonstrated a possible temporal association between psychological distress and onset, recurrence, and severity of psoriasis (Stewart et al., 2018), as well as the presence of high levels on negative affect in SSc patients (Leon et al., 2014), few studies have examined whether expressive suppression or cognitive reappraisal predicted well-being or psychological distress in dermatological patients. For example, Chung et al. (2018) have found that the suppression of depression predicted psychiatric co-morbidity in patients with chronic idiopathic urticaria, suggesting the important role of expressive suppression in skin diseases.

It is worth examining which aspects of positive functioning may reduce psychological distress and are associated with spiritual well-being in skin diseases patients. In this regard, sense of coherence (SOC) is a promising avenue of research. SOC refers to a person’s enduring feeling of confidence that the world and one’s life are comprehensible, manageable, and meaningful (Antonovsky, 1987). Although SOC was not associated with health satisfaction in individuals with SSc (Hyphantis et al., 2007b), this feeling of confidence negatively predicted general psychological distress symptoms (Hyphantis et al., 2007a) or depression (Matsuura et al., 2003) in these individuals.

Another important line of research has focused on positivity, which refers to a general tendency to evaluate self, life, and future with a positive outlook (Caprara et al., 2012). Individuals with psoriasis and low self-esteem were at risk for anxiety (Słomian et al., 2018), while optimism was the strongest predictor of HRQOL in psoriasis (Miniszewska et al., 2013). Moreover, optimism was also negatively associated with poor quality of life in individuals with SSc (Szramka-Pawlak et al., 2013).

In addition to emotion dysregulation and physical symptoms, research suggests the benefits of spiritual assessment and care for improving the quality of life of patients living with chronic dermatological diseases (e.g., Goldenberg and Jacob, 2015). In particular, spirituality is receiving increasing attention in research and clinical settings as an important strategy to cope with chronic illness (McGrady and Moss, 2018). It has been defined as the way “in which people can make sense of their lives, and feel whole, hopeful and peaceful even in the midst of life’s most serious challenges” (Brady et al., 1999, p. 418). Spiritual well-being (e.g., sense of meaning) was found to be clinically relevant to chronic patients and to have an impact on their HRQOL (Iani et al., 2018). Therefore, introducing measures of spiritual well-being into clinical research alongside existing measures may represent a promising approach (Joseph and Patterson, 2016). In this regard, scholars suggested considering spiritual well-being as an outcome after trauma (Park, 2017). Indeed, spiritual well-being is increasingly identified as not only a predictor of psychological adjustment but also an important aspect of well-being.

Investigating both positive and negative functioning aspects is important theoretically and clinically, since that it may indicate potential contributors to well-being and distress in individuals with skin diseases. The topics of positive clinical psychology (e.g., SOC, positivity) may have incremental validity in predicting clinical outcomes, so that positive functioning may be related to less psychological distress and to greater well-being (Joseph and Patterson, 2016). Although studies have been conducted on quality of life and psychological distress in individuals with psoriasis and SSc, little is known about the role of SOC, cognitive reappraisal, and positivity in influencing spiritual well-being and distress in these individuals.

According to this theoretical framework, the aim of the present study was to investigate whether positive (reappraisal, SOC, and positivity) and negative functioning (physical symptoms, type of disease, and expressive suppression) were associated with spiritual well-being and psychological distress. Based on research findings, we expected that: (1) SOC and positivity would be associated with spiritual well-being and psychological distress after controlling for different aspects of negative functioning; (2) skin-related symptoms and emotion dysregulation (i.e., suppression of expressive behavior) would be associated with psychological distress after controlling for SOC and positivity.

## Materials and Methods

### Participants and Procedure

A total of 192 consecutive individuals (112 women, 79 men, 1 unknown gender; *M*_*age*_ = 51.6 years, *SD* = 16.5) attending the Istituto Dermopatico dell’Immacolata (IDI-IRCCS, Italy) were enrolled in the study. Participants were recruited during their hospital stay for psoriasis or SSc. Eligible participants had a diagnosis of psoriasis or SSc by a board-certified dermatologist, were 18 years of age or more, had no mental disorders, did not undergo psychotherapy for at least 6 months in the last 3 years, and did not currently receive psychopharmacological treatment. Written consent was obtained from all participants after a detailed explanation of the study. Moreover, they were also informed about the voluntary nature of participation and the right to withdraw from the study at any time. The study was approved by the local Ethics Committee (number 46/CE/2016) and was carried out in accordance with the 1964 Helsinki Declaration.

### Measures

#### Independent Variables

We used different measures of positive functioning. The cognitive reappraisal from the Emotion Regulation Questionnaire (ERQ) was used to assess cognitive change that modifies the emotional impact of a situation (Balzarotti et al., 2010). Individuals reported the frequency with which they generally have experienced each item on a 7-point scale, from 1 (*strongly disagree*) to 7 (*strongly agree*). A sample item is “I control my emotions by changing the way I think about the situation I’m in.” Omega coefficient was 0.82. We also assessed comprehensibility/manageability and meaningfulness derived from the short form of the SOC scale (Barni and Tagliabue, 2005). Items are rated on a 7-point scale, with higher scores indicating greater SOC. Sample items are “Do you have very mixed-up feelings and ideas?” (comprehensibility/manageability) and “Do you have the feeling that you really don’t care about what is going on around you?” (reverse item, meaningfulness). Omega coefficients were 0.71 and 0.58 for comprehensibility/manageability and meaningfulness, respectively. The Positivity scale (Caprara et al., 2012) was used to assess the tendency to evaluate self, life, and future with a positive outlook. Items are measured on a 5-point scale (1 = *strongly disagree*, 5 = *strongly agree*), with higher scores reflecting greater positivity. A sample item is “I am satisfied with my life.” Omega coefficient was 0.82.

Besides the aspects of positive functioning, we used two measures of negative functioning. The symptoms scale of the Skindex-29 was used to measure skin-related symptoms (Abeni et al., 2002). Items are rated on a 5-point frequency scale (0 = *never*; 4 = *all the time*), with higher scores indicating higher levels of symptoms. A sample item is “My skin is sensitive.” Omega coefficient was 0.80. The Expressive suppression subscale of the ERQ was used to measure a response-focused strategy aimed at modifying the emotion-expressive behavior (Balzarotti et al., 2010). The response format was the same as for the cognitive reappraisal subscale. A sample item is “I keep my emotions to myself.” Omega coefficient for expressive suppression was 0.71.

#### Dependent Variables

Spiritual well-being was assessed with the Functional Assessment of Chronic Illness Therapy-Spiritual Well-Being Scale (FACIT-Sp: Rabitti et al., 2020), which measures faith, peace, and meaning. Items are rated on a 5-point frequency scale (0 = *not at all*; 4 = *very much*), with higher scores indicating greater spiritual well-being. A sample item is “I feel peaceful.” Omega coefficient was 0.83.

Psychological distress was measured with the 12-item General Health Questionnaire (Picardi et al., 2001). Items are rated on a 4-point scale (0 = *not at all*; 3 = *much more than usual*), with higher scores indicating more psychological distress. A sample item is “Have you recently felt constantly under strain?.” Omega coefficient was 0.89. Data on socio-demographic variables, clinical history, and other clinical characteristics (e.g., symptoms, duration of the disease, comorbidities, etc.) were obtained from medical records.

### Data Analysis

For assessing reliability we used the omega coefficient, which is more suitable than the Cronbach’s α (Dunn et al., 2014). We used *t*-test and chi-square to compare psychological and socio-demographic variables of clinical groups. Associations between variables were evaluated with Pearson’s *r*. Two hierarchical multiple regressions were conducted to determine whether spiritual well-being and psychological distress were accounted for by skin-related symptoms, type of disease, emotion regulation strategies, SOC, and positivity. The order of independent variables in hierarchical multiple regressions is guided by the purpose of the research (i.e., the specific questions to be answered; Cohen et al., 2003). Based on research findings and consistent with the hypotheses of the study, the independent variables were entered in the following order: skin-related symptoms, type of disease, emotion regulation, SOC, and positivity. The ratio of number of participants to number of predictor variables was just over 20:1. The diagnostics for multicollinearity revealed acceptable results (all VIF values <2). Occasional missing values were imputed by calculating, for each participant, the average score for each subscale and then replaced.

## Results

### Demographic and Psychological Variables

About 15% of eligible participants were excluded from the analyses because of significant missing data (*n* = 28, 14.6%). Individuals excluded due to missing data were older than those included (*M*_*age*_ 66.3 versus 49.9, *p* < 0.001), but gender (*X*^2^[1, *N* = 191] = 0.84, *p* = 0.361) and type of disease (*X*^2^[1, *N* = 192] = 1.62, *p* = 0.202) were similar. Sample characteristics are presented in Table 1. Individuals with psoriasis and SSc were similar in most sociodemographic and psychological features. Only exceptions were the prevalence of female participants with SSc (in accordance with epidemiological data). Individuals with SSc were older than individuals with psoriasis and scored higher on cognitive reappraisal. When age was included as a covariate, the significant difference in cognitive reappraisal disappeared.

**TABLE 1:** Demographic and psychological variables according to type of disease.

Variable	Total sampleMean (*SD*)	Psoriasis(*n* = 112)	Systemic sclerosis(*n* = 41)	*t* or *X*^2^	*p*
Age *M* (*SD*)	49.9 (15.7)	47.5 (15.7)	56.5 (13.6)	*t*(81.9) = −3.45	0.001
Gender *N* (%)				*X*^2^(1, *N* = 164) = 41.49	< 0.001
Male	70 (42.7)	69 (58.0)	1 (2.2)		
Female	94 (57.3)	50 (42.0)	44 (97.8)		
Symptoms	13.9 (6.4)	14.1 (6.5)	13.6 (6.3)	*t*(82.3) = 0.44	0.658
Reappraisal	27.6 (8.6)	26.7 (9.0)	29.9 (7.1)	*t*(100.0) = −2.40	0.018
Suppression	14.4 (6.1)	14.7 (6.3)	13.4 (5.5)	*t*(90.5) = 1.27	0.206
Comprehensibility/manageability	33.2 (7.9)	32.7 (8.3)	34.4 (6.5)	*t*(100.0) = −1.41	0.160
Meaningfulness	20.2 (4.8)	20.0 (5.0)	21.0 (4.2)	*t*(93.6) = −1.28	0.204
Positivity	29.7 (6.2)	29.5 (6.6)	30.4 (5.2)	*t*(98.8) = −0.96	0.338
Spiritual well-being	29.1 (8.5)	28.7 (8.7)	29.9 (8.2)	*t*(81.6) = −0.81	0.418
Psychological distress	14.9 (6.6)	14.9 (6.6)	14.6 (6.6)	*t*(69.4) = 0.28	0.776

### Correlations Among Variables

Correlations are provided in Table 2. The aspects of positive functioning (e.g., SOC, positivity, and spiritual well-being) and psychological distress were intercorrelated, suggesting that the higher the individual’s generalized resistance resources, the lower the perceived psychological distress. Positivity and SOC were positively associated with spiritual well-being and the latter was negatively associated – with a lower magnitude – with skin-related symptoms. Physical symptoms were also positively associated with expressive suppression and psychological distress. Of note, meaningfulness was the only specific aspect that was significantly associated with all other variables. Moreover, the aspects of SOC were the only variables that were highly correlated (*r* > ± 0.50) with both spiritual well-being and psychological distress.

**TABLE 2:** Relationships among variables.

	Symptoms	Reappraisal	Suppression	Meaningfulness	Comprehens./ manag.	Positivity	Spiritual well-being	Psychological distress
Symptoms	1							
Reappraisal	–0.10	1						
Suppression	0.20*	0.29***	1					
Meaningfulness	−0.31***	0.16*	−0.32***	1				
Comprehens./manag.	−0.28***	0.16*	–0.09	0.58***	1			
Positivity	−0.23**	0.23**	–0.12	0.56***	0.55***	1		
Spiritual well-being	−0.29***	0.21**	–0.12	0.56***	0.57***	0.66***	1	
Psychological distress	0.47***	−0.18*	0.30***	−0.55***	−0.53***	−0.41***	−0.47***	1

Comprehens./manag. = Comprehensibility/manageability (Soc). *p < 0.05; **p <0.01; ***p < 0.001.

### Regression Analyses

Table 3 reports the final regression models that describe the relationships of skin-related symptoms, type of disease, emotion regulation, SOC, and positivity with spiritual well-being and psychological distress (further details are provided in Supplementary Tables S1, S2). Since we did not find any effect on spiritual well-being and psychological distress due to gender, age, comorbidity, and disease duration, we excluded these variables from the analyses.

**TABLE 3:** Hierarchical regression analysis for variables predicting spiritual well-being and psychological distress: Model 5.

	*ΔR*^2^	*β*
**Dependent variable: Spiritual well-being**		
Step 1	0.08***	
Symptoms		–0.08
Step 2	0.00	
Type of disease		–0.01
Step 3	0.05*	
Reappraisal		0.06
Suppression		0.01
Step 4	0.29***	
Comprehensibility/manageability		0.21**
Meaningfulness		0.18*
Step 5	0.10***	
Positivity		0.42***
Total *R*^2^	0.53***	
**Dependent variable: Psychological distress**		
Step 1	0.22***	
Symptoms		0.27***
Step 2	0.00	
Type of disease		0.07
Step 3	0.10***	
Reappraisal		−0.15*
Suppression		0.19**
Step 4	0.16***	
Comprehensibility/manageability		−0.28***
Meaningfulness		−0.21*
Step 5	0.00	
Positivity		–0.03
Total *R*^2^	0.48	

*p < 0.05, **p < 0.01, ***p < 0.001.

#### Spiritual Well-Being

In the first step of the regression model, physical symptoms were associated with spiritual well-being (*R*^2^ = 0.08, *p* < 0.001). The addiction of type of disease in the second step did not reveal any significant association between type of disease and spiritual well-being (Δ*R*^2^ = 0.00, *ns*). In the third step, emotion regulation accounted for 5.2% of the variance in spiritual well-being, with a significant contribution of cognitive reappraisal (β = 0.24; *p* < 0.01). In the fourth step, SOC explained an additional 29% of the variance, with a significant contribution of both comprehensibility/manageability and meaningfulness (β = 0.35, *p* < 0.001 and β = 0.32, *p* < 0.001, respectively). In the fifth step, positivity also revealed a significant association with spiritual well-being (β = 0.42; *p* < 0.001) and accounted for an additional 10.5% of the variance. When all the independent variables were included, only comprehensibility/manageability, meaningfulness, and positivity were significantly associated with spiritual well-being, accounting for 53% of the variance.

#### Psychological Distress

In the first step of the regression model, skin-related symptoms were associated with psychological distress (β = 0.47; *p* < 0.001) and accounted for 22.1% of the variance. The addiction of type of disease in the second step did not reveal any significant association between type of disease and psychological distress (Δ*R*^2^ = 0.00, *ns*). In the third step, emotion regulation accounted for 9.7% of the variance in psychological distress, with a significant contribution of both cognitive reappraisal and expressive suppression (β = −3.44, *p* < 0.001 and β = 4.14, *p* < 0.001). Introducing SOC in the fourth step explained an additional 16.4% of the variance, with a significant contribution of both comprehensibility/manageability and meaningfulness (β = −3.88, *p* < 0.001 and β = −2.75, *p* < 0.01, respectively). The addiction of positivity in the fifth step did not reveal any significant association between positivity and psychological distress (Δ*R*^2^ = 0.00, *ns*). When all the independent variables were included, skin-related symptoms, and both different emotion regulation strategies as well as SOC dimensions were significantly associated with psychological distress, accounting for 48.2% of the variance.

## Discussion

The optimal clinical management of individuals with chronic medical diseases should include not only the attempt to reduce the burden of disease (e.g., impaired psychosocial functioning) but also to promote positive functioning (Joseph and Patterson, 2016). Clinicians’ and researchers’ awareness of the importance of positive aspects of psychological functioning has created a growing body of literature on dimensions such as spiritual well-being, SOC, and positivity. In the present study we investigated how aspects of positive and negative functioning are associated with psychological distress and spiritual well-being in individuals with chronic skin diseases.

Firstly, we expected that SOC and positivity would account for spiritual well-being and psychological distress after controlling for different aspects of negative functioning such as skin-related symptoms and expressive suppression. Our findings confirmed that positivity was particularly important to determine the extent to which spirituality can help individuals with skin diseases “to make sense of their lives, and feel whole, hopeful and peaceful even in the midst of a serious illness” (Bredle et al., 2011, 78). This result is consistent with a previous study with non-clinical samples in which prioritizing positivity was positively associated with positive emotions and satisfaction with life (Catalino et al., 2014). The influence of positivity on spiritual well-being is not surprising due to the fact that they were highly correlated. However, the magnitude of the relationship between positivity and spiritual well-being was not so large to indicate that these constructs are largely overlapping or analogous. In sum, positivity is an important and independent contributor to spiritual well-being in individuals with skin diseases.

Positivity was not associated with psychological distress in skin disease patients. This finding seems surprising given that previous studies showed the protective factor of positivity in sustaining cancer patients’ efforts to cope with severe illness (Caprara et al., 2016). The results of our study suggest that others personal resources, such as the ability to comprehend and manage the experience of the illness, better explain their role in reducing the effects of skin-related symptoms and expressive suppression on psychological distress in these patients.

In our study, both SOC subscales were associated with spiritual well-being. Our findings suggest that feelings of confidence that skin disease is comprehensible, manageable, and meaningful may help individuals in accepting their illness, achieving harmony, or even reassessing the existential meaning of their problematic conditions. This finding is worth noting because, to our knowledge, little research has examined this issue in individuals with psoriasis and in people with SSc.

Sense of coherence was also significantly associated with lower levels of psychological distress in our sample, suggesting that the more these individuals are able to make sense of and manage their illness, the less they experience distress. This result is consistent with previous studies showing that low levels of SOC were associated with general psychological distress symptoms in individuals with SSc (Hyphantis et al., 2007a). Our results can be interpreted through the lens of positive clinical psychology. A likely explanation is that SOC can serve as protective factor from negative mental health. Traditional clinical psychology has focused more on the assessment of psychological distress or disorders. However, positive psychology characteristics have shown incremental validity in predicting clinical distress (Wood and Johnson, 2016). Moreover, previous research has documented the potential protective power of positive human functioning in resistance to and recovery from illness, and the absence of positivity as vulnerability factor (Ryff and Singer, 1998; Iani et al., 2019). Consistent with this theoretical framework, our results show that positive functioning aspects, and especially SOC, may reduce the effects of skin-related symptoms and emotion dysregulation in influencing psychological distress in individuals with skin diseases.

In our second hypothesis, we expected that skin-related symptoms would be associated with psychological distress rather than spiritual well-being, after controlling for SOC and positivity. Our findings confirmed this hypothesis: the more skin-related symptoms patients are suffering from, the more likely spiritual well-being tends to remain stable. A possible explanation may be that the slow and progressive course of SSc and the relapsing nature of psoriasis make the patients experience their illness as distressing but not threatening their spiritual well-being. A comparison of this finding with previous research is not possible since, to date, very little is known about the associations between skin-related quality of life and spiritual well-being. The only study that investigated the links between similar constructs has shown that existential well-being predicted mental functioning in individuals with chronic skin diseases (Unterrainer et al., 2016). However, the authors used measurement scales (spirituality, quality of life) different from ours.

Skin-related symptoms were associated with psychological distress. This finding is not unexpected since previous research showed a strong relationship between HRQOL (physical symptoms) and psychiatric morbidity (Abeni et al., 2002; Sampogna et al., 2004b). Indeed, negative effects on health due to skin-related symptoms are associated with the degree to which individuals with skin diseases report minor psychiatric disorders. This result is consistent with previous studies showing that different aspects of poor skin-related quality of life, such as higher levels of fatigue and perceived helplessness (Evers et al., 2005) as well as defectiveness/shame and social isolation (Mizara et al., 2012), best predicted psychological distress in individuals with skin diseases.

As hypothesized, expressive suppression was not associated with spiritual well-being. Instead, higher levels of expressive suppression were positively correlated with psychological distress when controlling for SOC and positivity. This result suggests that individuals with skin diseases who modulate their expression of emotion report worse psychological functioning. Our finding is consistent with previous studies showing that expressive suppression predicted depression and anxiety symptoms in a community sample of women experiencing traumatic events (e.g., Moore et al., 2008). Although suppression of negative emotions is often used to decrease emotional experiencing, it paradoxically increases psychological distress and, therefore, may be considered a maladaptive emotion regulation strategy (Werner and Gross, 2010).

Finally, cognitive reappraisal was not related to spiritual well-being whereas it was negatively associated with psychological distress. The latter result shows that individuals with skin diseases who reduce negative emotions through cognitive change report less psychological symptoms. This finding is consistent with a previous study showing that the use of reappraisal predicted lower levels of psychological symptoms in college students (Brewer et al., 2016). Therefore, according to the literature (Werner and Gross, 2010), reappraisal can be considered an adaptive emotion regulation strategy.

### Limitations and Future Directions

This study has some limitations. First, the generalizability of our results is limited by the use of self-reported, cross-sectional data. Future longitudinal studies with larger samples would be required to establish causal links between variables and to generalize the results. Second, we did not consider physiological symptoms that characterize these conditions. Future studies might use disease biomarkers (e.g., cortisol, immune activity), together with self-report measures, to provide a multimodal assessment of well-being and psychological distress. Third, we did not compare patients with healthy controls. Future studies should investigate whether these psychological resources have a different role in clinical versus community samples.

Notwithstanding these limitations, this study represents an important step toward understanding the extent to which inner resources contribute to spiritual well-being in individuals with skin diseases over and above skin-related symptoms and emotion dysregulation. As such, it has implications for the promotion of spiritual well-being as well as the treatment of psychological distress in these individuals. Although some authors call for further research on the benefits of spiritual assessment and care for individuals with skin diseases (Goldenberg and Jacob, 2015), their spiritual needs are supported minimally or not at all in clinical practice. The highest levels of unmet needs in individuals with SSc are in the psychological/spiritual/existential domain (Rubenzik and Derk, 2009). The ability to have a comprehensive understanding of the illness, achieve a restoration of meaning in the experience of skin disease by providing an explanation for one’s suffering and illness, keep a positive outlook, and maintain self-worth might help individuals with skin diseases to adjust to illness, and clinicians to give better care to these individuals.

The findings of this study are of relevance in skin disease treatment and pave the way for further research on how SOC and positivity may reduce the effects of both skin-related symptoms and emotion dysregulation and facilitate spiritual well-being of individuals with skin diseases. These data provide preliminary support for the potential clinical utility of positive psychological factors in individuals with skin diseases and suggest that clinical or health psychologists may promote recovery following chronic skin diseases by focusing on psychological resources and spiritual well-being. Interventions aimed to enhance inner resources of these individuals and help them to find a meaning in their experience of skin disease might reduce psychological distress and improve spiritual well-being by restoring a more adaptive sense of self and the world. Indeed, there is evidence showing that individuals can receive substantial help in finding new meaning and purpose in life through expressive writing interventions, which may favor psychological growth in chronic disease (Cafaro et al., 2019) and play a role in enhancing treatment efficacy and quality of life (Paradisi et al., 2010). This study also suggests that clinical or health psychologists working with individuals with chronic skin diseases should have training in the spiritual dimensions of illness.

## Data Availability Statement

The raw data supporting the conclusions of this article will be made available by the authors, without undue reservation.

## Ethics Statement

The studies involving human participants were reviewed and approved by the Ethical Committee of IDI-IRCCS. The patients/participants provided their written informed consent to participate in this study.

## Author Contributions

LI designed the study, analyzed the data, and wrote the manuscript. RMQ and PP wrote the manuscript and collaborated in the editing of the final manuscript. A-RA and AS collaborated in the editing of the final manuscript. DA designed the study and collaborated in the editing of the final manuscript. All authors contributed to the article and approved the submitted version.

## Conflict of Interest

The authors declare that the research was conducted in the absence of any commercial or financial relationships that could be construed as a potential conflict of interest.
